# Use of induction of labour and emergency caesarean section and perinatal outcomes in English maternity services: A national hospital‐level study

**DOI:** 10.1111/1471-0528.17193

**Published:** 2022-06-13

**Authors:** Ipek Gurol‐Urganci, Jennifer Jardine, Fran Carroll, Alissa Frémeaux, Patrick Muller, Sophie Relph, Lara Waite, Kirstin Webster, Sam Oddie, Jane Hawdon, Tina Harris, Asma Khalil, Jan van der Meulen, National Maternity and Perinatal Audit Project Team, Megan Coe, George Dunn, Julia Langham, Dharmintra Pasupathy, Louise Thomas

**Affiliations:** ^1^ Department of Health Services Research and Policy London School of Hygiene and Tropical Medicine London UK; ^2^ Royal College of Obstetricians and Gynaecologists London UK; ^3^ Bradford Teaching Hospitals NHS Foundation Trust Bradford UK; ^4^ Royal Free London NHS Foundation Trust London UK; ^5^ Centre for Reproduction Research, Faculty of Health and Life Sciences De Montfort University Leicester UK; ^6^ Fetal Medicine Unit, Department of Obstetrics and Gynaecology St George’s University Hospitals NHS Foundation Trust London UK; ^7^ Vascular Biology Research Centre, Molecular and Clinical Sciences Research Institute St George’s University of London London UK

**Keywords:** caesarean, induction, intervention, labour, pregnancy, quality, stillbirth

## Abstract

**Objective:**

To assess the association between hospital‐level rates of induction of labour and emergency caesarean section, as measures of ‘practice style’, and rates of adverse perinatal outcomes.

**Design:**

National study using electronic maternity records.

**Setting:**

English National Health Service.

**Sample:**

Hospitals providing maternity care to women between April 2015 and March 2017.

**Main outcome measures:**

Stillbirth, admission to a neonatal intensive care unit and babies receiving mechanical ventilation.

**Results:**

Among singleton term births, the risk of stillbirth was 0.15%, the risk of admission to a neonatal intensive care unit was 5.4% and the risk of mechanical ventilation 0.54%. There was considerable between‐hospital variation in the rate of induction of labour (minimum 17.5%, maximum 40.7%) and in the rate of emergency caesarean section (minimum 5.6%, maximum 17.1%). Women who gave birth in hospitals with a higher rate of induction of labour had better perinatal outcomes. For each 5%‐point increase in induction, there was a decrease in the risk of term stillbirth of 9% (OR 0.91, 95% CI 0.85–0.97) and a decrease in the risk of mechanical ventilation of 14% (OR 0.86, 95% CI 0.79–0.94). There was no significant association between hospital‐level induction of labour rates and neonatal unit admission at term (*p* > 0.05). There was no significant association between hospital‐level rates of emergency caesarean section and adverse perinatal outcomes (all with *p* > 0.05).

**Conclusions:**

There is considerable between‐hospital variation in the use of induction of labour and emergency caesarean section. Hospitals with a higher rate of induction had a lower risk of adverse birth outcomes. A similar association was not found for caesarean section.

## Abstract 2

**Linked article**: This article is commented on by Eric Jauniaux, pp. 1907 in this issue. To view this minicommentary visit https://doi.org/10.1111/1471‐0528.17277

This article includes Author Insights, a video abstract available at: https://vimeo.com/bjogabstracts/authorinsights17193

## Graphical abstract

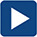

## 1 INTRODUCTION

The care that maternity services provide to women giving birth needs to be finely balanced between supporting the physiological process of birth and intervening when required. This is the fundamental principle underpinning the clinical guidelines on care of women during pregnancy and birth, all aiming to ensure that giving birth is a safe and joyful experience, and that women and their families are treated with dignity and respect.^1^, ^2^

At the same time, there is ample evidence that maternity care varies widely between and within countries.^3^, ^4^ For example, in England, Scotland and Wales, the National Maternity and Perinatal Audit (NMPA), a national initiative to assess and improve the quality of maternity services, found that the rate of induction of labour varied between 16% and 44%, and that the overall rate of caesarean section varied between 17% and 35%, in singleton babies born at term in 149 hospital organisations of the National Health Service (NHS) that provided maternity services between April 2016 and March 2017.^5^

This between‐hospital variation reflects the lack of consensus about the indications for interventions such as induction of labour and emergency caesarean section. Positions in this debate are often based on arguments that focus either on the safety of childbirth or on the women’s experience.^6^ Some organisations supporting families are concerned that an emphasis on numerical targets related to safety may lead to poorer birth experiences, and to increases in the rates of induction of labour and caesarean section.^7^ Conversely, a recent high‐profile independent review of potentially avoidable harm in births occurring at an NHS hospital in England between 2000 and 2019 reported, after having reviewed the first 250 cases, that ‘there was a culture … to keep caesarean section rates low, because this was perceived as the essence of good maternity care’.^8^

In response to this debate, we have investigated the association of the rates of induction of labour and emergency caesarean section in each hospital in England with the risks of stillbirth, admission to a neonatal intensive care unit and the use of mechanical ventilation in babies born at term. We considered these two intervention rates as complementary measures of a hospital's culture, or more accurately ‘practice style’, with the rate of induction of labour reflecting how hospitals provide proactive care for women with term pregnancies and with the rate of emergency caesarean section reflecting how hospitals respond to acute situations that may need immediate preventive action or rescue.

## 2 METHODS

### 2.1 Data sources

We used a patient‐level data set compiled by the NMPA, based on records of each birth from data routinely collected in the maternity information systems (MIS) used by NHS hospitals to record care throughout pregnancy, and linked with data from the Hospital Episode Statistics (HES), the database that contains administrative data for all admissions to NHS hospitals.^5^, ^9^ The MIS databases include data items that typically cover antenatal booking through to birth and immediate postnatal care, entered by midwives and support staff in the antenatal clinic or labour ward. Although there were 20 different systems in use, each of which collects slightly different information, in sometimes different formats, there was sufficient similarity between systems to allow a single data set to be developed from which comparative measures can be derived.

This NMPA data set was also linked to the National Neonatal Research Dataset (NNRD), which contains information on admissions to neonatal care.^10^ Information was available on births between 1 April 2015 and 31 March 2017. The resulting linked data set, including 131 NHS hospitals, or more precisely NHS ‘hospital trusts’, which are NHS organisations with one or more sites providing secondary health services in a geographical area, with 1 253 847 births, captures approximately 94% of the births that occurred in England during the study period.^5^, ^10^ Further details about the data sets and linkage processes are available elsewhere.^5^, ^10^ Details of the data sets used to derive each variable in the analyses are available in Table S1.

### 2.2 Population

This study examined the association between hospital‐level rates of induction of labour and emergency caesarean section and the patient‐level risk of adverse perinatal outcomes in singleton pregnancies at term. Births were eligible for inclusion if they occurred between 1 April 2015 and 31 March 2017 in an NHS hospital providing maternity care in England. Overall, we included only those hospitals in the analyses where at least 70% of birth records within a hospital had information about the specific intervention and outcome. In addition, for each analysis of a specific association between a hospital‐level intervention rate and a perinatal adverse outcome, we only included births with complete information on both the specific intervention and the outcome measure being studied.

### 2.3 Perinatal outcomes, interventions and maternal and pregnancy characteristics

The adverse perinatal outcome measures were stillbirth, according to the NMPA data set, and admission to a neonatal intensive care unit at term and mechanical ventilation of the baby, according to the NNRD.^10^ These outcomes cover three of the eight core outcomes recommended by the Core Outcome Measures in Effectiveness Trials (COMET) initiative.^11^ It should be noted that the recommended core outcomes include ‘death of the baby’, defined as ‘intrapartum/neonatal/perinatal death’, whereas we included all antepartum and intrapartum stillbirth, irrespective of gestational age. Antepartum stillbirth was included because we consider it to be a relevant outcome when studying the association between the use of interventions and perinatal outcomes. We did not include neonatal death or perinatal death other than stillbirth because these outcomes were not fully covered in our linked data sets.

Information about induction of labour, emergency caesarean section (defined as a caesarean section that is not planned and/or prelabour), was obtained from the NMPA data set.^5^

Information about maternal characteristics, including age, obstetric history (parity and previous caesarean birth), body mass index (BMI) and comorbidities, including pre‐eclampsia/eclampsia, pre‐existing hypertensive disease and pre‐existing or gestational diabetes, was available in the NMPA data set. Women were assumed not to have a comorbid condition if relevant diagnostic codes were not present.^12^ If information about a woman’s obstetric history was missing in the birth record, a ‘look‐back’ approach in HES was used where all previous records for the woman since 2000 in English NHS hospitals were considered.^13^ Maternal ethnicity was coded using the Office for National Statistics categorisation system from the 2001 Census, collapsed into five groups: white, South‐Asian, black, mixed and other (including Chinese).^14^ Socio‐economic status was evaluated using the Index of Multiple Deprivation (IMD), an area‐level measure of deprivation identified by the woman’s recorded postcode in the NMPA data set, and grouped into quintiles according to the national distribution.^13^, ^15^

### 2.4 Statistical analysis

First, we determined for each hospital the rate of induction of labour and the rate of emergency caesarean section. In a second step, we included these hospital‐level rates of induction of labour and emergency caesarean section as risk factors in multilevel regression models, with stillbirth, admission to a neonatal intensive care unit or mechanical ventilation as outcomes.

The multilevel models were used to test whether differences in the hospital‐level intervention rates were associated with the risk of an adverse outcome using odds ratios. A *p*‐value lower than 0.05 was considered to indicate a statistically significant association. Given that stillbirth, admission to a neonatal intensive care unit and mechanical ventilation of the baby are rare events from an epidemiological perspective, odds ratios can be interpreted as measures of relative risk.^16^

The hospital‐level rates of induction of labour and emergency caesarean section were recoded, so that the odds ratios estimated by the models represent relative differences in the risk (or more precisely in the ‘odds’) of the perinatal adverse outcomes associated with a 5%‐point increase in these intervention rates to make the results easier to interpret. In other words, the hospital‐level intervention rates were multiplied by 20 so that a 5%‐point increase was transformed into a one‐unit increase (20 × 5% = 1).

We also estimated odds ratios with adjustment for individual maternal characteristics, including age (grouped as <20, 20–34, 35–39 and ≥40 years of age), parity and previous caesarean birth, BMI (grouped as <18.5, 18.5–24.9, 25.0–29.9, 30.0–34.9, 35.0–39.9 and ≥ 40 kg/m^2^), pre‐eclampsia, pre‐existing hypertensive disease, pre‐existing or gestational diabetes, maternal ethnicity and socio‐economic deprivation, which were chosen as they are known to be strongly associated with adverse perinatal outcomes.^17^, ^18^ For each maternal characteristic, missing values were assigned to a separate ‘missing’ category, so that all included births with complete information on the specific intervention and outcome measures could be retained in the regression analyses.

We carried out two supplementary analyses. First, we investigated the association between the hospital intervention rates and the proportion of term babies born before 39 completed weeks of gestation. We performed this supplementary analysis because an increased use of induction of labour may be associated with an increase in the proportion of babies born before 39 weeks of gestation.^19^, ^20^ A second supplementary analysis was carried out, only including primiparous women, given that our previous work has highlighted that parity is a considerably stronger risk factor than other risk factors for adverse perinatal outcomes.^18^

All analyses were performed in Stata 16 (StataCorp, College Station, TX, USA).

### 2.5 Patient and public involvement

This study was motivated by the public debate triggered by the publication of the Ockenden report in 2020 and the consultation for the draft guideline on the induction of labour of the National Institute of Health and Care Excellence (NICE).^8^, ^21^ Women and families were not directly involved in the design of the study, the data analysis or the interpretation of the results.

### 2.6 Role of the funding source

The NMPA is commissioned by the Healthcare Quality Improvement Partnership (HQIP, www.hqip.org.uk) as part of the National Clinical Audit and Patient Outcomes Programme, and is funded by NHS England and the Scottish and Welsh governments. Neither HQIP nor the funders had any involvement in designing the study, collecting, analysing and interpreting the data, writing the report or the decision to submit the article for publication.

## 3 RESULTS

The data set included 131 hospitals and 1 131 719 singleton term births. The number of hospitals included in the analyses varied between 92 and 109, depending on the specific intervention and the outcome measure being analysed (Figure S1). Hospitals that were excluded for data quality were more likely to have a lower number of births (fewer than 4000 per annum) than the hospitals that were included. The characteristics of women in the included and excluded units were similar. A detailed description of the maternal characteristics of the included and excluded births can be found in Table S2. Overall, the risk of stillbirth at term was 0.15% (in 101 hospitals and 935 053 births), the risk of admission to a neonatal intensive care unit was 5.4% (in 112 hospitals and 998 933 births) and the risk of mechanical ventilation of the baby was 0.54% (in 112 hospitals and 998 933 births).

**FIGURE 1:**
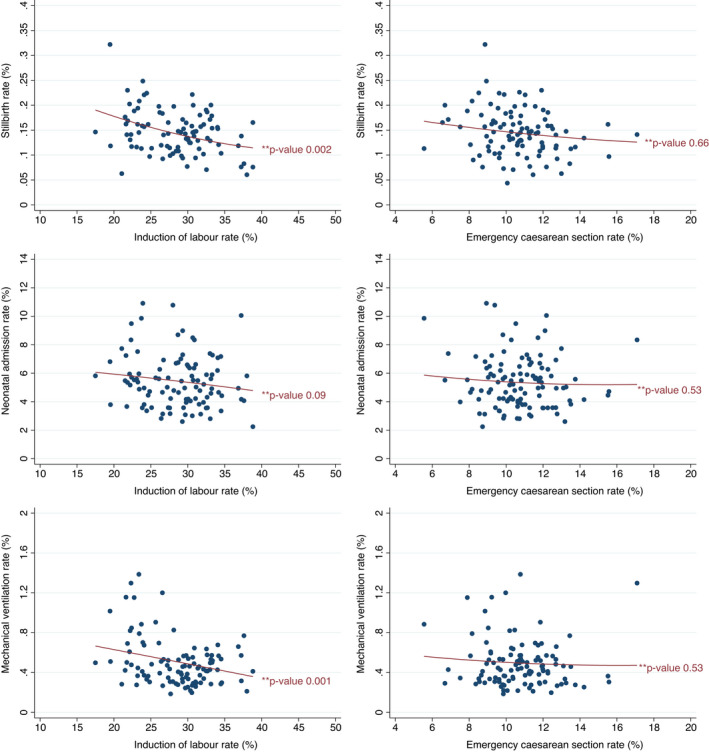
Risks observed in hospitals (dots) and predicted risks (line) of the three perinatal outcomes according to the rates of induction of labour and emergency caesarean section in the hospitals

Figure 1 presents plots of the observed risks of the three perinatal outcomes according to the rates of induction of labour and emergency caesarean section in the hospitals, as well as the predicted risks. These plots show the between‐hospital variation in the rate of induction of labour (minimum 17.5%, maximum 40.7%, IQR 24.6%–32.1%) and in the rate of emergency caesarean section (minimum 5.6%, maximum 17.1%, IQR 9.4%–11.8%).

Figure 1 also demonstrates that the risk of adverse perinatal outcomes tends to be lower in hospitals with higher intervention rates, but that only the associations between the rate of induction of labour and rates of stillbirth and mechanical ventilation were statistically significant (*p* = 0.002 and *p* = 0.001, respectively).

Table 1 demonstrates a similar pattern of results with adjustment for maternal characteristics. These adjusted results show that a 5%‐point increase in the rate induction of labour at a hospital was associated with a 9% reduction (95% CI 3%–15%, corresponding to an OR of 0.91) in the risk of stillbirth in term pregnancies and a 14% reduction (95% CI 6%–21%, corresponding to an OR of 0.86) in the risk of a baby requiring mechanical ventilation. No corresponding association was found between the rates of emergency caesarean section and the risks of stillbirth, admission to a neonatal intensive care unit or mechanical ventilation of the baby. Full model results are presented in Table S3.

**TABLE 1:** Odds ratios corresponding to a 5%‐point increase in the rate of induction of labour or in the rate of emergency caesarean section

	OR (95% CI)	*p*‐value	OR (95% CI)adjusted for maternal characteristics^a^	*p*‐value
Stillbirth rate				
Induction of labour (92 hospitals with 842 737 births)	0.89 (0.83–0.94)	<0.001	0.91 (0.85–0.97)	0.002
Emergency caesarean section (99 hospitals with 905 081 births)	0.86 (0.73–1.01)	0.07	0.96 (0.82–1.13)	0.66
Neonatal intensive care unit admission				
Induction of labour (101 hospitals with 839 754 births)	0.94 (0.88–1.01)	0.11	0.94 (0.87–1.01)	0.09
Emergency caesarean section (109 hospitals with 964 353 births)	0.94 (0.79–1.13)	0.52	0.94 (0.79–1.13)	0.53
Mechanical ventilation rate				
Induction of labour (101 hospitals with 839 754 births)	0.87 (0.80–0.94)	0.001	0.86 (0.79–0.94)	0.001
Emergency caesarean section (109 hospitals with 964 353 births)	0.92 (0.74–1.15)	0.47	0.93 (0.74–1.17)	0.53

^a^ Maternal characteristics included age, maternal ethnicity, BMI, socio‐economic status, parity, previous caesarean birth, maternal hypertensive disease and maternal diabetes.

In the first supplementary analysis, we found evidence that hospitals with higher induction of labour rates had more term babies born before 39 completed weeks of gestation, without and with adjustment for maternal characteristics (Table S4). In the second supplementary analysis, including only primiparous women, the pattern of results was very similar (Table S5), but the association between induction of labour rates and stillbirth was no longer statistically significant.

## 4 DISCUSSION

### 4.1 Main findings

Hospitals with a higher rate of induction of labour had lower risks of stillbirth and mechanical ventilation of babies born after 37 completed weeks of gestation. There was no evidence of an association between the rate of emergency caesarean section at a hospital and the risk of adverse perinatal outcome.

### 4.2 Strengths and limitations

The key strengths of our study are its size and design. First, this study uses data from births in at least 92 of the 134 hospitals providing maternity services between 2015 and 2017 in the NHS in England. Second, unlike many other studies in this area that report solely on stillbirth, we could report on a wider range of perinatal outcomes, such as admission to a neonatal intensive care unit and the mechanical ventilation of the baby. Third, the completeness of the data from the hospitals included in the analyses was high, so that meaningful adjustments could be made at individual patient level for a wide range of maternal characteristics.

A first limitation is that we did not have information about the indications for the interventions, which limits the exploration of the reasons for the observed variation. Second, a higher rate of induction of labour may be linked to other differences in the care delivered to women with pregnancies beyond 37 weeks of gestation. For example, we did not have data about screening for fetal growth restriction, fetal monitoring or one‐on‐one continuous care from a primary midwife through pregnancy and birth. Therefore, residual confounding cannot be fully excluded, despite adjustments for the most important maternal risk factors.^18^, ^22^ However, residual confounding does not explain our findings because both the rates of interventions and the rates of adverse perinatal outcomes are likely to be higher in women with an increased risk profile. Third, not all hospitals could be included because of a high level of missing data about the specific intervention and outcome. The characteristics of the women in the included and excluded units were similar, and therefore it is unlikely that a particular pattern of ‘missingness’ can explain our results (Table S2).

### 4.3 Interpretation (in light of other evidence)

The variation observed in the induction of labour and emergency caesarean section rates suggests that there are marked differences in the practice style of hospitals providing maternity services in the English health service. However, only higher rates of induction were found to be associated with a slightly lower risk of adverse perinatal outcomes, both when all births were considered and when only births in primiparous women were included. As expected, the results for admission to a neonatal intensive care unit were very similar to those for mechanical ventilation, but this association was not found to be significant. This may reflect that admission to a neonatal intensive care unit reflects a wider range of less specific adverse outcomes than those that trigger an induction of labour.

At a risk of over‐interpreting the results, one could argue that the results for induction of labour indicate that hospitals with a practice style that includes a lower threshold for the induction of labour in women with pregnancies at term seem to have better outcomes, but that the results for emergency caesarean section suggest that there is no – or at best little – evidence that the risks of poor perinatal outcomes are linked to how teams of midwives and obstetricians in English NHS hospitals use caesarean section in emergency situations.

This reduction in the risk of adverse birth outcomes with higher hospital‐level rates of induction may come at a price. We found that an increased rate in the induction of labour increased the rate of births before 39 weeks of gestation. This is a potential concern as long‐term studies of childhood outcomes have shown that neurocognitive and health outcomes may improve with each week of gestation up to 40 weeks of gestation.^23^, ^24^ Also, it has been shown that an emergency caesarean section in the first pregnancy is associated with a shorter length of gestation, increased rate of repeat caesarean section and increased rate of admission to a neonatal intensive care unit in the next pregnancy.^25^

### 4.4 Clinical and research implications

Intervention rates in maternity services are difficult to target. For example, the reported rates for caesarean section in European countries vary widely: from 16.5% in Norway to 35.4% in Italy.^26^ There is no consensus about the optimal population‐level caesarean section rate.^27^ Similarly, an overview of four major international guidelines found considerable variation in recommendations on the timing and, as a consequence, the overall frequency of induction of labour,^28^ but there is growing evidence for the clinical and cost‐effectiveness of induction of labour beyond 41 weeks of gestation.^29^

This lack of consensus is not surprising, because midwives and obstetricians are guided by imprecise evidence about relatively low risks of serious outcomes when they help women to choose where and how to give birth to their baby. In line with the longstanding dichotomies in this debate, a focus on safety may lead to doing ‘too much, too soon’, whereas a focus on the women’s experience may lead to doing ‘too little, too late’.^1^

Our results provide important background information for the independent review of cases of potentially avoidable harm in births in specific hospitals in England.^8^, ^30^, ^31^ These reviews often have little or no access to comparative data from births without adverse outcomes or from hospitals with different intervention rates or with different risks of adverse perinatal outcome. It has been argued that these reviews, without being nested in larger epidemiological studies, such as the one described in this article, are only appropriate for hypothesis generation, but they are frequently used to make high‐profile recommendations.^32^

In the UK, the national guidelines for induction of labour have just been updated.^33^ This followed a national debate regarding the appropriateness of draft recommendations, which recommended an offer of induction of labour at 41 completed weeks of gestation to all women, and from 39 completed weeks of gestation to women with uncomplicated pregnancies who are at increased risk of stillbirth because of their clinical profile.^21^ Women’s advocacy and support groups, as well as organisations representing healthcare professionals, expressed concern that an increased use of induction of labour would harm maternal experience, and that ‘singling out’ women based on their age, ethnic background or BMI may be considered discriminatory, if not fully backed up by evidence.^34^ These recommendations have therefore not been included in the final version of the national guidelines, and more research is recommended to establish if, and at what gestational age, induction of labour should be offered.

This research needs to include large numbers given that the risk of adverse outcomes is low, which makes it unlikely that randomised controlled trials can be designed with adequate statistical power.^35^ Non‐randomised studies, using routinely collected clinical data, provide an alternative approach to fill this evidence gap, provided that the level of data completeness and quality is sufficiently high so that adjustments can be made for differences in the characteristics of the women who do and do not have the intervention. Therefore, our study reiterates the need to have more complete and accurate maternity data at a national level than are currently available.^36^

## 5 CONCLUSION

There was considerable between‐hospital variation in the use of induction of labour and emergency caesarean section in singleton term births. Hospitals with a higher rate of induction had a lower risk of adverse birth outcomes but a similar association was not found for emergency caesarean section. This suggests that a more proactive practice style with an increased use of induction of labour, rather than an increased use of caesarean section in emergency situations, seems to be linked to safer childbirth at term. Our results also demonstrate that independent reviews investigating concerns about the safety of maternity services in a specific hospital might benefit from being nested in larger epidemiological studies.

### National Maternity and Perinatal Audit Project Team members

Fran Carroll, Megan Coe, George Dunn, Alissa Frémeaux, Ipek Gurol‐Urganci, Tina Harris, Jane Hawdon, Jennifer Jardine, Amar Karia, Asma Khalil, Julia Langham, Jan van der Meulen, Patrick Muller, Sam Oddie, Dharmintra Pasupathy, Sophie Relph, Louise Thomas, Lara Waite and Kirstin Webster.

### CONFLICT OF INTERESTS

All authors, apart from JvdM, are or have been partially or wholly funded by the Healthcare Quality Improvement Partnership for their contribution to the submitted work. All authors also declare no financial relationships with any organisation that might have an interest in the submitted work in the previous 3 years. The authors report no other relationships or activities that could appear to have influenced the submitted work. Completed disclosure of interests form available to view online as supporting information.

### DISSEMINATION TO PARTICIPANTS AND RELATED PATIENT AND PUBLIC COMMUNITIES

We disseminate results through patient organisations and representative groups of women giving birth in the UK.

### AUTHOR CONTRIBUTIONS

JvdM, IGU, PM and AK conceived the study. IGU conducted the analysis. All authors contributed to the interpretation of the results. JJ wrote the first draft of the article, which was reviewed and revised by all authors. JvdM edited the final version. IGU is the guarantor and confirms that this manuscript is an accurate reflection of the results.

### DETAILS OF ETHICS APPROVAL

This study used data routinely collected in clinical care to evaluate service provision and performance, and therefore individual consent was not sought. Permission to access the data was provided by the NHS Health Research Authority Confidentiality Advisory Group, approval number 16/CAG/0058. The data were used to evaluate service provision and performance and therefore the study was exempt from ethical review by the NHS Health Research Authority.

## Supporting information


Appendix S1

Appendix S2

Data S1

Data S2

Data S3

Data S4

Data S5

Data S6

Data S7

Data S8

Data S9

Data S10

Data S11

Data S12

Data S13

## Data Availability

The data are available for further research and service evaluation after approval from the data controller, which is the Healthcare Quality Improvement Partnership.
