# Supplementary material for: Use of induction of labour and emergency caesarean section and perinatal outcomes in English maternity services: A national hospital‐level study
Source: BJOG. 2022 Jun 13;129(11):1899–906. doi: 10.1111/1471-0528.17193 (PMC9543153; doi:10.1111/1471-0528.17193)
Supplement: Supplementary file 1 — Appendix S1 [file BJO-129-1899-s001.pdf]

Supplementary Figure 1: Study flowchart

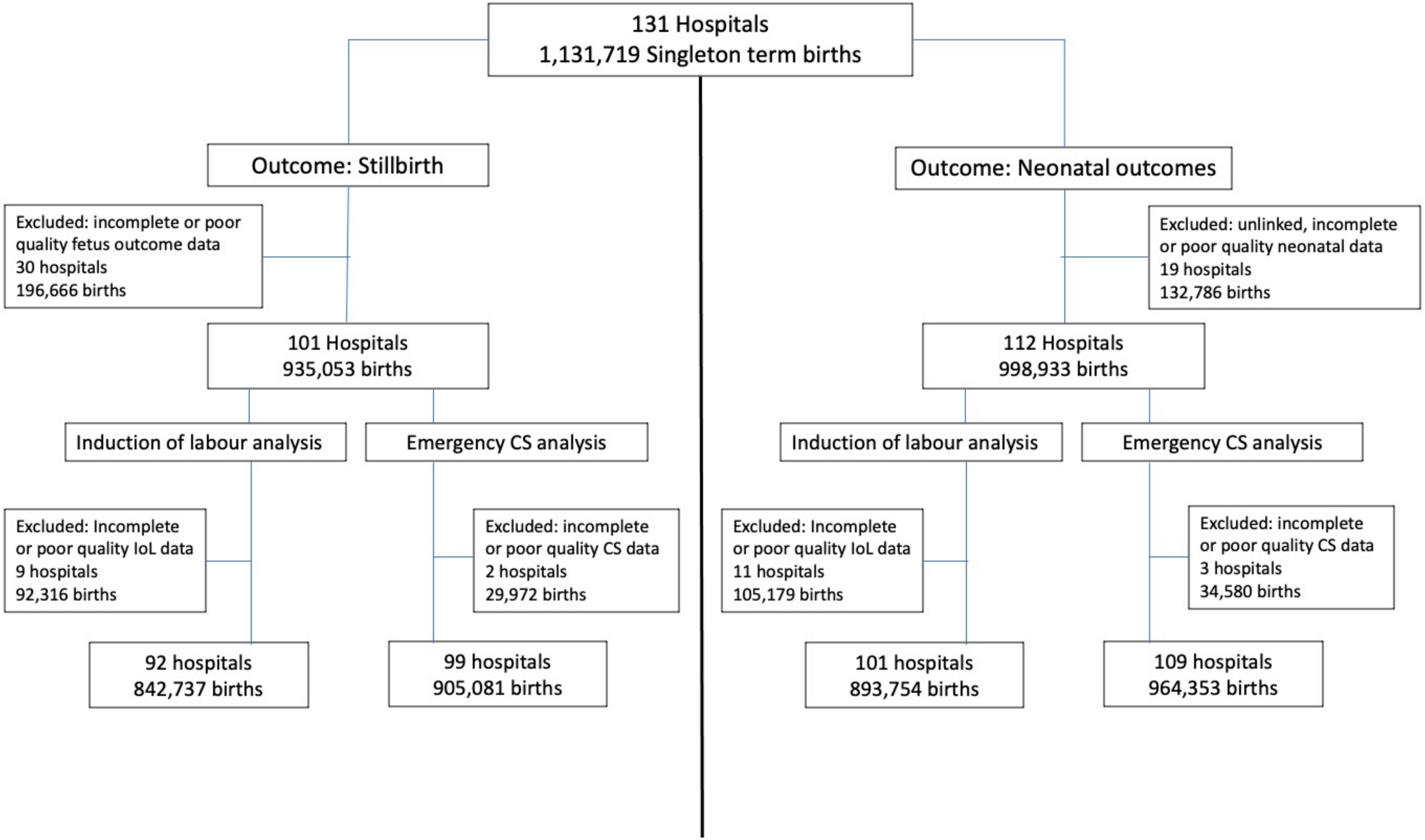

Supplementary Figure 2: Hospital level interventions and outcomes – primiparous women

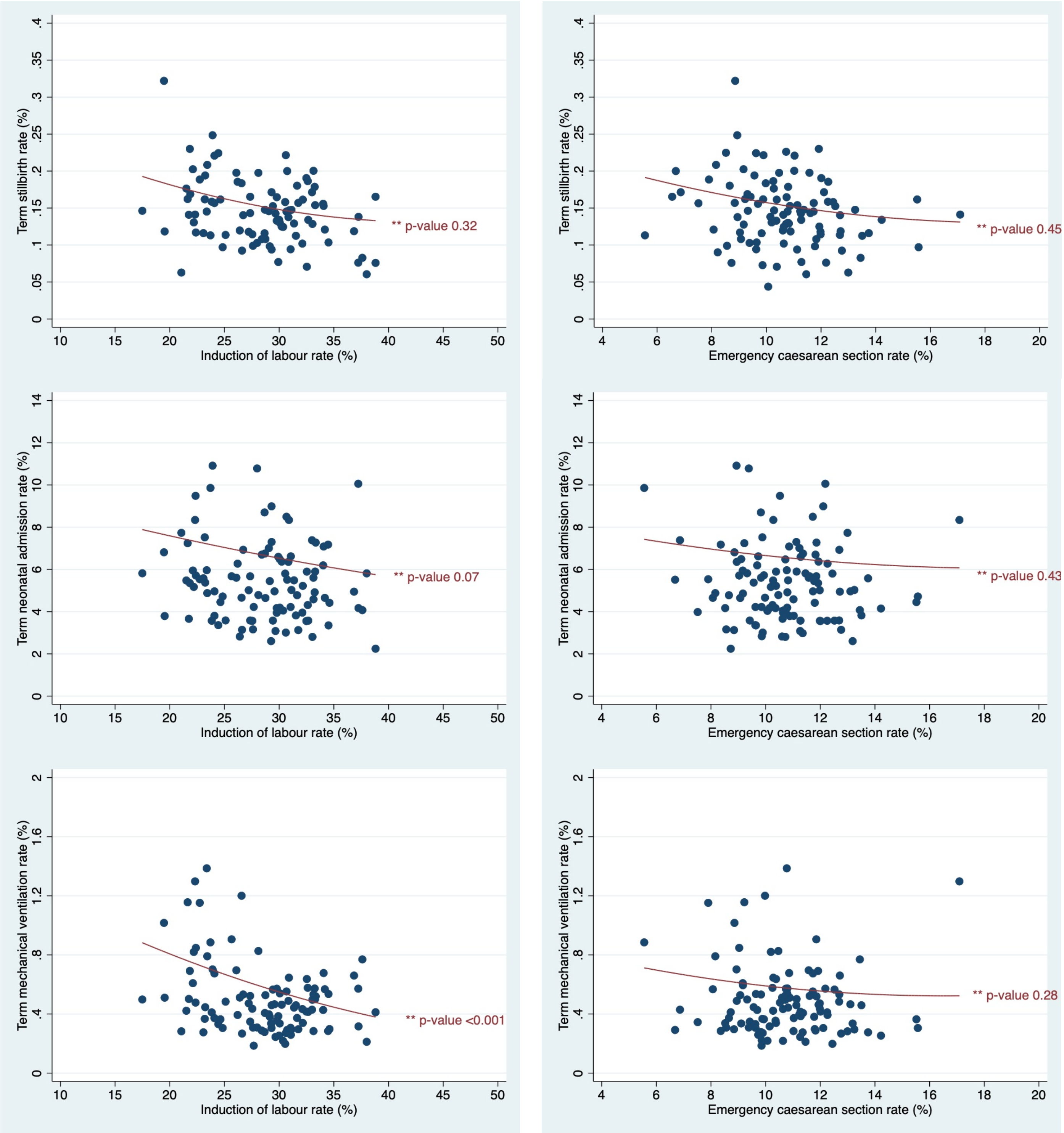

* Predictions of the multilevel logistic regression model described in the Methods section with adjustment for maternal characteristics.

** P value of the association between the perinatal outcome and the hospital-level intervention rate with adjustment for maternal characteristics.
