# Supplementary material for: Use of induction of labour and emergency caesarean section and perinatal outcomes in English maternity services: A national hospital‐level study
Source: BJOG. 2022 Jun 13;129(11):1899–906. doi: 10.1111/1471-0528.17193 (PMC9543153; doi:10.1111/1471-0528.17193)
Supplement: Supplementary file 2 — Appendix S2 [file BJO-129-1899-s012.docx]

**Supplementary Table 1**: Variable specifications, sources and categories used within the NMPA-NNRD dataset

| **Variable** | **Data Source** | **Details** |
| --- | --- | --- |
| **Outcomes** |  |  |
| Stillbirth | Primary: MIS  Secondary: HES | Defined in the UK as the birth of a baby with no signs of life at or after 24 completed weeks of gestation. This includes both antepartum and intrapartum stillbirth. |
| Admission to neonatal unit | NNRD | Any recorded admission to a neonatal unit (not including transitional care units, for example on postnatal wards) |
| Mechanical ventilation | NNRD | This refers to a baby who is intubated with an endotracheal tube and attached to a ventilator. This is also known as ‘invasive ventilation’. This does not include non-invasive ventilation (CPAP, BiPAP, high-flow oxygen via nasal cannulae). |
| **Interventions** |  |  |
| Emergency caesarean section | Primary: MIS  Secondary: HES | Birth recorded as emergency caesarean section in the mode of birth field |
| Induction of labour | MIS | Onset of labour recorded as by induction of labour |
| **Characteristics** |  |  |
| Maternal age | Primary: MIS  Secondary: HES | Maternal age at time of birth. Grouped into under 20, 20-34, 35-39, 40 or older |
| Obstetric history | Primary: MIS  Secondary: HES | Grouped into three categories: nulliparous; multiparous with previous caesarean; multiparous without previous caesarean |
| Body mass index | MIS | Grouped using WHO categories (<18.5kg/m2, 18.5-24.9, 25.0-29.9, 30-34.9, 35.0-39.9, 40 or over) |
| Pre-eclampsia/eclampsia | HES | O14, O15 |
| Pre-existing hypertensive disease | HES | O10, O11, I10-I15 |
| Pre-existing or gestational diabetes | HES | O24, E10-E14 |
| Ethnic group | Primary: HES  Secondary: MIS | Categorised into White, S Asian, Black, Mixed, Other |
| Socioeconomic group | Primary: MIS  Secondary: HES | Index of Multiple Deprivation (IMD) associated with women’s recorded postcode at time of birth, grouped into quintiles |
| HES – Hospital Episode Statistics; MIS – Maternity Information Systems; NNRD – National Neonatal Research Database | | |

**Supplementary Table 2a:** Descriptive statistics for excluded and included hospitals and births (induction of labour analyses)

|  | **Stillbirth cohort** | | **Neonatal outcomes cohorts** | |
| --- | --- | --- | --- | --- |
|  | **Included N(%)** | **Excluded N(%)** | **Included N(%)** | **Excluded N(%)** |
| **Number of hospitals** | 92 | 39 | 101 | 30 |
| **Hospital size per year** |  |  |  |  |
| Less than 2500 | 10 (10.9) | 12 (30.8) | 14 (13.9) | 8 (26.7) |
| 2500-3999 | 22 (23.9) | 14 (35.9) | 25 (24.8) | 11 (36.7) |
| 4000-5999 | 40 (43.5) | 9 (23.1) | 41 (40.6) | 8 (26.7) |
| More than 6000 | 20 (21.7) | 4 (10.3) | 21 (20.8) | 3 (10.0) |
| **Hospital type*** |  |  |  |  |
| OU only | 15 (16.3) | 12 (30.8) | 20 (19.8) | 7 (23.3) |
| OU and AMU | 49 (53.3) | 17 (43.6) | 50 (49.5) | 16 (53.3) |
| OU and FMU | 8 (8.7) | 3 (7.7) | 10 (9.9) | 1 (3.3) |
| OU, AMU and FMU | 20 (21.7) | 6 (15.4) | 21 (20.8) | 5 (16.7) |
| FMU only | - | 1 (2.6) | - | 1 (3.3) |
| **Number of births** | 842,737 | 288,982 | 893,754 | 237,965 |
| **Maternal age (years)** |  |  |  |  |
| <20 | 26690 (3.2) | 8840 (3.1) | 28642 (3.2) | 6888 (2.9) |
| 20-34 | 628932 (74.6) | 207073 (71.7) | 665405 (74.5) | 170600 (71.7) |
| 35-39 | 145774 (17.3) | 50729 (17.6) | 153246 (17.1) | 43257 (18.2) |
| ≥40 | 32750 (3.9) | 11777 (4.1) | 34478 (3.9) | 10049 (4.2) |
| Missing | 8591 (1.0) | 10563 (3.7) | 11983 (1.3) | 7171 (3.0) |
| **Obstetric history** |  |  |  |  |
| Primiparous | 348858 (41.4) | 117760 (40.7) | 368548 (41.2) | 98070 (41.2) |
| Multiparous with no previous CS | 364875 (43.3) | 122456 (42.4) | 388605 (43.5) | 98726 (41.5) |
| Multiparous with previous CS | 114223 (13.6) | 36074 (12.5) | 120823 (13.5) | 29474 (12.4) |
| Missing | 14781 (1.8) | 12692 (4.4) | 15778 (1.8) | 11695 (4.9) |
| **Maternal ethnic group** |  |  |  |  |
| White | 588165 (69.8) | 214710 (74.3) | 629812 (70.5) | 173063 (72.7) |
| South Asian | 100831 (12.0) | 20668 (7.2) | 100472 (11.2) | 21027 (8.8) |
| Black | 36879 (4.4) | 14012 (4.8) | 38133 (4.3) | 12758 (5.4) |
| Mixed | 14217 (1.7) | 5108 (1.8) | 14831 (1.7) | 4494 (1.9) |
| Other stated | 34154 (4.1) | 8925 (3.1) | 35027 (3.9) | 8052 (3.4) |
| Missing | 68491 (8.1) | 25559 (8.8) | 75479 (8.4) | 18571 (7.8) |
| **Index of Multiple Deprivation** |  |  |  |  |
| Quintile 1= most deprived | 218265 (25.9) | 68942 (23.9) | 237599 (26.6) | 49608 (20.8) |
| 2 | 181142 (21.5) | 60347 (20.9) | 191624 (21.4) | 49865 (21.0) |
| 3 | 150004 (17.8) | 51214 (17.7) | 156955 (17.6) | 44263 (18.6) |
| 4 | 128819 (15.3) | 49078 (17.0) | 136399 (15.3) | 41498 (17.4) |
| Quintile 5 = least deprived | 113234 (13.4) | 44086 (15.3) | 117967 (13.2) | 39353 (16.5) |
| Missing | 51273 (6.1) | 15315 (5.3) | 53210 (6.0) | 13378 (5.6) |

*OU: Obstetric unit, AMU: Alongside midwifery unit, FMU: Freestanding midwifery unit

**Supplementary Table 2b:** Descriptive statistics for excluded and included hospitals and births (Emergency caesarean section analyses)

|  | **Stillbirth cohort** | | **Neonatal outcomes cohorts** | |
| --- | --- | --- | --- | --- |
|  | **Included N(%)** | **Excluded N(%)** | **Included N(%)** | **Excluded N(%)** |
| **Number of hospitals** | 99 | 32 | 109 | 22 |
| **Hospital size per year** |  |  |  |  |
| Less than 2500 | 10 (10.1) | 12 (37.5) | 14 (12.8) | 8 (36.4) |
| 2500-3999 | 26 (26.3) | 10 (31.3) | 30 (27.5) | 6 (27.3) |
| 4000-5999 | 42 (42.4) | 7 (21.9) | 43 (39.4) | 6 (27.3) |
| More than 6000 | 21 (21.2) | 3 (9.4) | 22 (20.2) | 2 (9.1) |
| **Hospital type*** |  |  |  |  |
| OU only | 20 (20.2) | 7 (21.9) | 24 (22.0) | 3 (13.6) |
| OU and AMU | 51 (51.5) | 15 (46.9) | 54 (49.5) | 12 (54.5) |
| OU and FMU | 8 (8.1) | 3 (9.4) | 10 (9.2) | 1 (4.5) |
| OU, AMU and FMU | 20 (20.2) | 6 (18.8) | 21 (19.3) | 5 (22.7) |
| FMU only | - | 1 (3.1) | - | 1 (4.5) |
| **Number of births** | 905,081 | 226,638 | 964,353 | 167,366 |
| **Maternal age (years)** |  |  |  |  |
| <20 | 29149 (3.2) | 6381 (2.8) | 31126 (3.2) | 4404 (2.6) |
| 20-34 | 676379 (74.7) | 159626 (70.4) | 718223 (74.5) | 117782 (70.4) |
| 35-39 | 155275 (17.2) | 41228 (18.2) | 165125 (17.1) | 31378 (18.7) |
| ≥40 | 34952 (3.9) | 9575 (4.2) | 37256 (3.9) | 7271 (4.3) |
| Missing | 9326 (1) | 9828 (4.3) | 12623 (1.3) | 6531 (3.9) |
| **Obstetric history** |  |  |  |  |
| Primiparous | 375563 (41.5) | 91055 (40.2) | 398227 (41.3) | 68391 (40.9) |
| Multiparous with no previous CS | 391677 (43.3) | 95654 (42.2) | 419433 (43.5) | 67898 (40.6) |
| Multiparous with previous CS | 121470 (13.4) | 28827 (12.7) | 129392 (13.4) | 20905 (12.5) |
| Missing | 16371 (1.8) | 11102 (4.9) | 17301 (1.8) | 10172 (6.1) |
| **Maternal ethnic group** |  |  |  |  |
| White | 638200 (70.5) | 164675 (72.7) | 686063 (71.1) | 116812 (69.8) |
| South Asian | 104879 (11.6) | 16620 (7.3) | 104644 (10.9) | 16855 (10.1) |
| Black | 39216 (4.3) | 11675 (5.2) | 40641 (4.2) | 10250 (6.1) |
| Mixed | 14984 (1.7) | 4341 (1.9) | 15772 (1.6) | 3553 (2.1) |
| Other stated | 36119 (4.0) | 6960 (3.1) | 37076 (3.8) | 6003 (3.6) |
| Missing | 71683 (7.9) | 22367 (9.9) | 80157 (8.3) | 13893 (8.3) |
| **Index of Multiple Deprivation** |  |  |  |  |
| Quintile 1= most deprived | 238543 (26.4) | 48664 (21.5) | 256445 (26.6) | 30762 (18.4) |
| 2 | 192764 (21.3) | 48725 (21.5) | 204704 (21.2) | 36785 (22) |
| 3 | 159272 (17.6) | 41946 (18.5) | 167851 (17.4) | 33367 (19.9) |
| 4 | 138553 (15.3) | 39344 (17.4) | 148447 (15.4) | 29450 (17.6) |
| Quintile 5 = least deprived | 120482 (13.3) | 36838 (16.3) | 129418 (13.4) | 27902 (16.7) |
| Missing | 55467 (6.1) | 11121 (4.9) | 57488 (6.0) | 9100 (5.4) |

*OU: Obstetric unit, AMU: Alongside midwifery unit, FMU: Freestanding midwifery unit

**Supplementary Table 3a:** Multilevel logistic regression models (induction of labour)

|  | **Stillbirths**  **OR (95%CI), p-value** | | **Neonatal admissions**  **OR (95%CI), p-value** | | **Mechanical ventilation**  **OR (95%CI), p-value** | |
| --- | --- | --- | --- | --- | --- | --- |
| **Number of hospitals** | 92 | | 101 | | 101 | |
| **Number of births** | 842,737 | | 839,754 | | 839,754 | |
|  |  |  |  |  |  |  |
| **Induction of labour** | 0.91 (0.85,0.97) | 0.002 | 0.94 (0.87,1.01) | 0.09 | 0.86 (0.79,0.94) | 0.001 |
|  |  |  |  |  |  |  |
| **Maternal age (years)** |  |  |  |  |  |  |
| <20 | 0.82 (0.58,1.18) | 0.29 | 0.93 (0.88,0.98) | 0.01 | 0.95 (0.81,1.12) | 0.55 |
| 20-34 | Reference | | Reference | | Reference | |
| 35-39 | 1.29 (1.12,1.49) | <0.001 | 1.10 (1.08,1.13) | <0.001 | 1.09 (1.01,1.17) | 0.03 |
| ≥40 | 1.25 (0.95,1.64) | 0.11 | 1.18 (1.13,1.24) | <0.001 | 1.11 (0.97,1.28) | 0.14 |
| Missing | 1.29 (0.75,2.19) | 0.36 | 1.25 (1.12,1.38) | <0.001 | 1.67 (1.23,2.26) | 0.001 |
| **Obstetric history** |  |  |  |  |  |  |
| Primiparous | Reference | | Reference | | Reference | |
| Multiparous with no previous CS | 0.80 (0.70,0.90) | <0.001 | 0.57 (0.56,0.58) | <0.001 | 0.66 (0.62,0.71) | <0.001 |
| Multiparous with previous CS | 0.73 (0.61,0.87) | 0.001 | 0.80 (0.78,0.82) | <0.001 | 0.86 (0.78,0.93) | <0.001 |
| Missing | 0.94 (0.61,1.45) | 0.78 | 0.78 (0.73,0.84) | <0.001 | 1.03 (0.83,1.28) | 0.78 |
| **Maternal BMI (kg/m^2^)** |  |  |  |  |  |  |
| Underweight (<18.5) | 1.11 (0.75,1.64) | 0.61 | 0.92 (0.86,0.99) | 0.02 | 0.89 (0.72,1.09) | 0.25 |
| Ideal weight (18.5-24.9) | Reference | | Reference | | Reference | |
| Overweight (25.0-29.9) | 1.44 (1.24,1.67) | <0.001 | 1.19 (1.17,1.22) | <0.001 | 1.15 (1.06,1.24) | <0.001 |
| Grade I obese (30·0-34.9) | 1.61 (1.34,1.93) | <0.001 | 1.30 (1.26,1.34) | <0.001 | 1.35 (1.23,1.48) | <0.001 |
| Grade II obese (35.0-39.9) | 2.03 (1.61,2.57) | <0.001 | 1.51 (1.45,1.58) | <0.001 | 1.64 (1.45,1.85) | <0.001 |
| Grade II obese (≥40·0) | 2.89 (2.20,3.80) | <0.001 | 1.70 (1.61,1.79) | <0.001 | 1.98 (1.71,2.30) | <0.001 |
| Missing | 1.25 (1.04,1.50) | 0.02 | 1.24 (1.20,1.29) | <0.001 | 1.38 (1.24,1.53) | <0.001 |
| **Comorbidities** |  |  |  |  |  |  |
| Pre-eclampsia/eclampsia | 2.47 (1.85,3.30) | <0.001 | 1.94 (1.84,2.06) | <0.001 | 1.59 (1.33,1.90) | <0.001 |
| Pre-existing hypertensive diseases | 0.37 (0.12,1.15) | 0.09 | 1.39 (1.25,1.54) | <0.001 | 1.14 (0.82,1.58) | 0.43 |
| Pre-existing or gestational diabetes | 0.86 (0.68,1.08) | 0.20 | 1.71 (1.65,1.77) | <0.001 | 1.21 (1.08,1.35) | 0.001 |
| Missing | 1.06 (0.85,1.33) | 0.60 | 1.02 (0.98,1.06) | 0.32 | 0.97 (0.86,1.09) | 0.61 |
| **Maternal ethnic group** |  |  |  |  |  |  |
| White | Reference | | Reference | | Reference | |
| South Asian | 1.74 (1.50,2.03) | <0.001 | 1.02 (0.98,1.05) | 0.36 | 0.99 (0.90,1.09) | 0.85 |
| Black | 1.51 (1.20,1.90) | <0.001 | 0.98 (0.93,1.03) | 0.37 | 1.03 (0.91,1.17) | 0.61 |
| Mixed | 1.37 (0.93,2.02) | 0.11 | 0.87 (0.81,0.94) | <0.001 | 0.97 (0.79,1.20) | 0.8 |
| Other stated | 1.07 (0.80,1.43) | 0.64 | 0.88 (0.83,0.92) | <0.001 | 0.83 (0.72,0.97) | 0.02 |
| Missing | 0.99 (0.79,1.23) | 0.91 | 1.01 (0.98,1.05) | 0.48 | 1.13 (1.02,1.25) | 0.02 |
| **Index of Multiple Deprivation** |  |  |  |  |  |  |
| Quintile 1= most deprived | Reference | | Reference | | Reference | |
| 2 | 0.78 (0.67,0.92) | 0.002 | 0.91 (0.89,0.94) | <0.001 | 0.97 (0.89,1.05) | 0.45 |
| 3 | 0.82 (0.69,0.97) | 0.02 | 0.89 (0.86,0.92) | <0.001 | 0.89 (0.81,0.97) | 0.01 |
| 4 | 0.81 (0.68,0.98) | 0.03 | 0.88 (0.86,0.91) | <0.001 | 0.87 (0.79,0.96) | 0.01 |
| Quintile 5 = least deprived | 0.68 (0.55,0.83) | <0.001 | 0.84 (0.81,0.87) | <0.001 | 0.80 (0.72,0.90) | <0.001 |
| Missing | 0.89 (0.70,1.13) | 0.32 | 0.95 (0.91,0.99) | 0.03 | 1.02 (0.90,1.16) | 0.73 |

**Supplementary Table 3b:** Multilevel logistic regression models (emergency caesarean section)

|  | **Stillbirths**  **OR (95%CI), p-value** | | **Neonatal admissions**  **OR (95%CI), p-value** | | **Mechanical ventilation**  **OR (95%CI), p-value** | |
| --- | --- | --- | --- | --- | --- | --- |
| **Number of hospitals** | 99 | | 109 | | 109 | |
| **Number of births** | 905,081 | | 964,354 | | 964,354 | |
|  |  |  |  |  |  |  |
| **Caesarean section** | 0.96 (0.82,1.13) | 0.66 | 0.94 (0.79,1.13) | 0.53 | 0.93 (0.74,1.17) | 0.53 |
|  |  |  |  |  |  |  |
| **Maternal age (years)** |  |  |  |  |  |  |
| <20 | 0.82 (0.58,1.15) | 0.26 | 0.94 (0.90,0.99) | 0.02 | 1.01 (0.87,1.18) | 0.86 |
| 20-34 | Reference | | Reference | | Reference | |
| 35-39 | 1.31 (1.14,1.50) | <0.001 | 1.11 (1.08,1.13) | <0.001 | 1.10 (1.02,1.18) | 0.01 |
| ≥40 | 1.28 (0.99,1.67) | 0.06 | 1.19 (1.14,1.24) | <0.001 | 1.13 (0.99,1.30) | 0.07 |
| Missing | 1.32 (0.79,2.21) | 0.30 | 1.19 (1.07,1.31) | 0.001 | 1.60 (1.19,2.16) | 0.002 |
| **Obstetric history** |  |  |  |  |  |  |
| Primiparous | Reference | | Reference | | Reference | |
| Multiparous with no previous CS | 0.78 (0.69,0.88) | <0.001 | 0.57 (0.56,0.58) | <0.001 | 0.66 (0.62,0.70) | <0.001 |
| Multiparous with previous CS | 0.74 (0.62,0.88) | 0.001 | 0.81 (0.78,0.83) | <0.001 | 0.86 (0.79,0.94) | 0.001 |
| Missing | 0.95 (0.63,1.43) | 0.80 | 0.79 (0.74,0.85) | <0.001 | 0.96 (0.78,1.18) | 0.68 |
| **Maternal BMI (kg/m^2^)** |  |  |  |  |  |  |
| Underweight (<18.5) | 1.11 (0.76,1.63) | 0.60 | 0.91 (0.86,0.98) | 0.01 | 0.87 (0.71,1.07) | 0.19 |
| Ideal weight (18.5-24.9) | Reference | | Reference | | Reference | |
| Overweight (25.0-29.9) | 1.45 (1.26,1.67) | <0.001 | 1.20 (1.17,1.22) | <0.001 | 1.16 (1.08,1.25) | <0.001 |
| Grade I obese (30·0-34.9) | 1.57 (1.32,1.88) | <0.001 | 1.30 (1.26,1.34) | <0.001 | 1.37 (1.25,1.50) | <0.001 |
| Grade II obese (35.0-39.9) | 2.04 (1.62,2.56) | <0.001 | 1.51 (1.45,1.57) | <0.001 | 1.64 (1.46,1.85) | <0.001 |
| Grade II obese (≥40·0) | 2.88 (2.21,3.77) | <0.001 | 1.68 (1.60,1.77) | <0.001 | 2.00 (1.73,2.32) | <0.001 |
| Missing | 1.23 (1.04,1.47) | 0.02 | 1.24 (1.20,1.28) | <0.001 | 1.38 (1.25,1.52) | <0.001 |
| **Comorbidities** |  |  |  |  |  |  |
| Pre-eclampsia/eclampsia | 2.47 (1.87,3.27) | <0.001 | 1.97 (1.86,2.08) | <0.001 | 1.60 (1.35,1.90) | <0.001 |
| Pre-existing hypertensive diseases | 0.47 (0.18,1.26) | 0.14 | 1.41 (1.27,1.56) | <0.001 | 1.21 (0.88,1.65) | 0.24 |
| Pre-existing or gestational diabetes | 0.84 (0.67,1.06) | 0.13 | 1.72 (1.66,1.77) | <0.001 | 1.20 (1.07,1.33) | 0.001 |
| Missing | 1.07 (0.86,1.33) | 0.54 | 1.03 (1.00,1.07) | 0.08 | 0.99 (0.89,1.11) | 0.92 |
| **Maternal ethnic group** |  |  |  |  |  |  |
| White | Reference | | Reference | | Reference | |
| South Asian | 1.77 (1.52,2.06) | <0.001 | 1.02 (0.99,1.05) | 0.19 | 1.01 (0.92,1.11) | 0.84 |
| Black | 1.53 (1.22,1.91) | <0.001 | 0.98 (0.94,1.03) | 0.47 | 1.01 (0.89,1.15) | 0.84 |
| Mixed | 1.34 (0.91,1.97) | 0.14 | 0.89 (0.83,0.96) | 0.002 | 0.98 (0.80,1.21) | 0.87 |
| Other stated | 1.06 (0.79,1.41) | 0.70 | 0.87 (0.83,0.92) | <0.001 | 0.84 (0.72,0.97) | 0.02 |
| Missing | 1.03 (0.83,1.27) | 0.79 | 1.01 (0.98,1.05) | 0.45 | 1.16 (1.06,1.28) | 0.002 |
| **Index of multiple deprivation** |  |  |  |  |  |  |
| Quintile 1= most deprived | Reference | | Reference | | Reference | |
| 2 | 0.81 (0.69,0.94) | 0.01 | 0.92 (0.89,0.94) | <0.001 | 0.99 (0.91,1.07) | 0.74 |
| 3 | 0.81 (0.68,0.96) | 0.01 | 0.89 (0.86,0.92) | <0.001 | 0.92 (0.84,1.00) | 0.05 |
| 4 | 0.81 (0.68,0.97) | 0.02 | 0.88 (0.85,0.91) | <0.001 | 0.89 (0.81,0.98) | 0.02 |
| Quintile 5 = least deprived | 0.66 (0.54,0.81) | <0.001 | 0.83 (0.80,0.86) | <0.001 | 0.82 (0.74,0.91) | <0.001 |
| Missing | 1.03 (0.83,1.28) | 0.80 | 0.96 (0.92,1.00) | 0.03 | 1.03 (0.91,1.15) | 0.68 |

**Supplementary Table 4**. Odds ratios for birth before 39 weeks corresponding to a 5% increase in the induction of labour or in the emergency caesarean section rate

|  | **Odds ratio  (95% confidence interval)** | **p-value** | **Odds ratio (95% confidence interval) adjusted for maternal characteristics*** | **p-value** |
| --- | --- | --- | --- | --- |
| **Birth before 39 weeks** |  |  |  |  |
| Induction of labour  (105 hospitals with 921,393 births) | 1.06 (1.03 – 1.10) | <0.001 | 1.07 (1.04 – 1.10) | <0.001 |
| Emergency caesarean section  (116 hospitals with 1,015,405 births) | 1.03 (0.95 – 1.11) | 0.64 | 1.07 (0.99 – 1.15) | 0.09 |

* Maternal characteristics included age, obstetric history, BMI, pre-eclampsia/eclampsia, pre-existing hypertensive disease, pre-existing or gestational diabetes, ethnicity and socioeconomic status.

**Supplementary Table 5.** Odds ratios corresponding to a 5%-point increase in the induction of labour or the emergency caesarean section rate – primiparous women only

|  | **Odds ratio  (95% confidence interval)** | **p-value** | **Odds ratio (95% confidence interval) adjusted for maternal characteristics*** | **p-value** |
| --- | --- | --- | --- | --- |
| **Stillbirth rate** |  |  |  |  |
| Induction of labour  (92 hospitals with 348,858 births) | 0.92 (0.82-1.02) | 0.12 | 0.95 (0.85 – 1.05) | 0.32 |
| Emergency caesarean section  (99 hospitals with 375,563 births) | 0.83 (0.64 – 1.08) | 0.16 | 0.90 (0.69 – 1.17) | 0.45 |
| **Neonatal admission rate** |  |  |  |  |
| Induction of labour  (101 hospitals with 368,549 births) | 0.92 (0.85 – 1.00) | 0.06 | 0.93 (0.85 – 1.01) | 0.07 |
| Emergency caesarean section  (109 hospitals with 398,228 births) | 0.91 (0.74 – 1.11) | 0.34 | 0.92 (0.75 – 1.13) | 0.43 |
| **Mechanical ventilation rate** |  |  |  |  |
| Induction of labour  (101 hospitals with 368,549 births) | 0.82 (0.74 – 0.90) | <0.001 | 0.82 (0.75 - 0.91) | <0.001 |
| Emergency caesarean section  (109 hospitals with 398,228 births) | 0.86 (0.66 – 1.10) | 0.24 | 0.87 (0.67 – 1.12) | 0.28 |

* Maternal characteristics included age, BMI, pre-eclampsia/eclampsia, pre-existing hypertensive disease, pre-existing or gestational diabetes, ethnicity and socioeconomic status.
